# MLL-AF4 and a murinized pSer-variant thereof are turning on the nucleolar stress pathway

**DOI:** 10.1186/s13578-022-00781-y

**Published:** 2022-04-25

**Authors:** Anna Lena Siemund, Thomas Hanewald, Eric Kowarz, Rolf Marschalek

**Affiliations:** Institute of Pharmaceutical Biology/DCAL, Goethe-University of Frankfurt, Biocenter, Max-von-Laue-Straße 9, 60438 Frankfurt/Main, Germany

**Keywords:** *MLL-r* acute leukemia, MLL, AF4, Fusion proteins, pSer domain, SL1, Ribosome biogenesis

## Abstract

**Background:**

Recent pathomolecular studies on the MLL-AF4 fusion protein revealed that the murinized version of MLL-AF4, the MLL-Af4 fusion protein, was able to induce leukemia when expressed in murine or human hematopoietic stem/progenitor cells (Lin et al. in Cancer Cell 30:737–749, 2016). In parallel, a group from Japan demonstrated that the pSer domain of the AF4 protein, as well as the pSer domain of the MLL-AF4 fusion is able to bind the Pol I transcription factor complex SL1 (Okuda et al. in Nat Commun 6:8869, 2015). Here, we investigated the human MLL-AF4 and a pSer-murinized version thereof for their functional properties in mammalian cells. Gene expression profiling studies were complemented by intracellular localization studies and functional experiments concerning their biological activities in the nucleolus.

**Results:**

Based on our results, we have to conclude that MLL-AF4 is predominantly localizing inside the nucleolus, thereby interfering with Pol I transcription and ribosome biogenesis. The murinized pSer-variant is localizing more to the nucleus, which may suggest a different biological behavior. Of note, AF4-MLL seems to cooperate at the molecular level with MLL-AF4 to steer target gene transcription, but not with the pSer-murinized version of it.

**Conclusion:**

This study provides new insights and a molecular explanation for the described differences between hMLL-hAF4 (not leukemogenic) and hMLL-mAf4 (leukemogenic). While the human pSer domain is able to efficiently recruit the SL1 transcription factor complex, the murine counterpart seems to be not. This has several consequences for our understanding of t(4;11) leukemia which is the most frequent leukemia in infants, childhood and adults suffering from *MLL-r* acute leukemia.

**Supplementary Information:**

The online version contains supplementary material available at 10.1186/s13578-022-00781-y.

## Introduction

*MLL-r* leukemia is diagnosed in 5–10% of all acute leukemia patients, and the spectrum of *MLL* fusion partners has increased over the last 30 years of research to more than 100 [3]. The most frequent translocation in ALL is the chromosomal translocation t(4;11)(q21;q23) which represents overall about 57% of all patient cases. In this particular translocation, the two genes *MLL* (*KMT2A*) and *AF4* (*AFF1*) are fused in a balanced recombination event to cause the generation of the two fusion genes *MLL-AF4* and *AF4-MLL*, respectively.

Both MLL and AF4 wildtype protein complexes have important functions in mammalian cells. The MLL wildtype protein complex is known to confer active chromatin marks on target gene promotors which counteracts Polycomb repressor complexes and enables target gene transcription [4–6]. The AF4 complex [7, 8], also termed “super-elongation complex” (reviewed in Ref. [9]), is responsible for transcriptional elongation by recruiting several other histone methlyl transferases and using the P-TEFb kinase to convert the promoter-proximal arrested POL II into elongating POL II [8, 10, 11]. The action of both wildtype protein complexes is basically confering stable and tissue-specific gene expression.

In the past decades, researchers have tried to dissect the role of the MLL-AF4 and AF4-MLL fusion proteins. Most studies have failed to demonstrate oncogenic behavior of the MLL-AF4 fusion protein in vitro or in vivo (summarized in Ref. [13]), except two studies. Lin et al. was able to recapitulate leukemia development in mice when using a partially murinized h*MLL-mAf4* expression construct in hematopoietic target cells [1]. However, they failed with the full-human counterpart, *MLL-AF4*, to convincingly create leukemia. Our own study has shown that the AF4-MLL fusion protein is indispensable for leukemia onset, as the onset of leukemia in murine hematopoietic stem/progenitor cells was observed only in the presence of *AF4-MLL* or both fusion genes, but never with *MLL-AF4* alone [13]. However, the penetrance was only around 35% which was partly due to the difficulties in getting efficient packaging of the overlong retroviral vectors constructs, because in the transplantation experiment only 1 in 10,000 hematopoietic stem/precuror cells could be transduced with MLL-AF4 and 1 in 1000 with AF4-MLL. On the other hand, other transcription factors (e.g. RUNX1) may exhibit complementing functions to substitute the missing *MLL-AF4* allele in our experiments [14]. Based on our todays knowledge, the AF4-MLL fusion protein exhibits a chromatin opening functions which help MLL-AF4 to execute the activation of downstream target genes, but also the many other transcription factors that are already expressed in the stem cell compartment [12, 15].

It was therefore not so much surprising that the use of the CRISPR/CAS9 technology which allows per se to generate only balanced chromosomal translocations was sufficient to cause the onset of leukemia in human cord blood cells [16].

In order to understand the role of these fusion proteins, a novel cornerstone was added by the Yokoyama group [2]. They unravelled the molecular interaction of the SL1 complex with the pSer domain of AF4 or MLL-AF4 fusion protein. They also dissected the pSer domain at the functional level [17]. SL1 represents a transcription factor complex that is composed by TAF_I_12, TAF_I_A, TAF_I_B, TAF_I_C, TAF_I_D and TBP. SL1 is—together with UBF—required for Pol I transcription of the ribosomal precursor RNA (45S) that is subsequently processed into 28S, 18S and 5.8S rRNA [18–22]. These rRNA molecules are then used to build up ribosomes inside of the nucleolus, and—after transfer to the cytosol—to execute protein biosynthesis.

Therefore, we got interested in investigating these novel findings and to find a rational explanation for the inability of human *MLL-AF4* to cause leukemia in mammalian cells, while the murinized version *MLL-Af4* does. To dissect this problem we designed a partially murinized version of human *MLL-AF4* by replacing the human pSer domain with sequences of the murine counterpart. This novel construct, *MLL-AF4m*, was used along with *MLL-AF4* and *AF4-MLL* to perform all subsequent experiments in order to gain new insights into the functional mechanism exerted by these fusion proteins.

## Results

### Construction of transgenes and establishment of stable and inducible cell lines

**Fig. 1:**
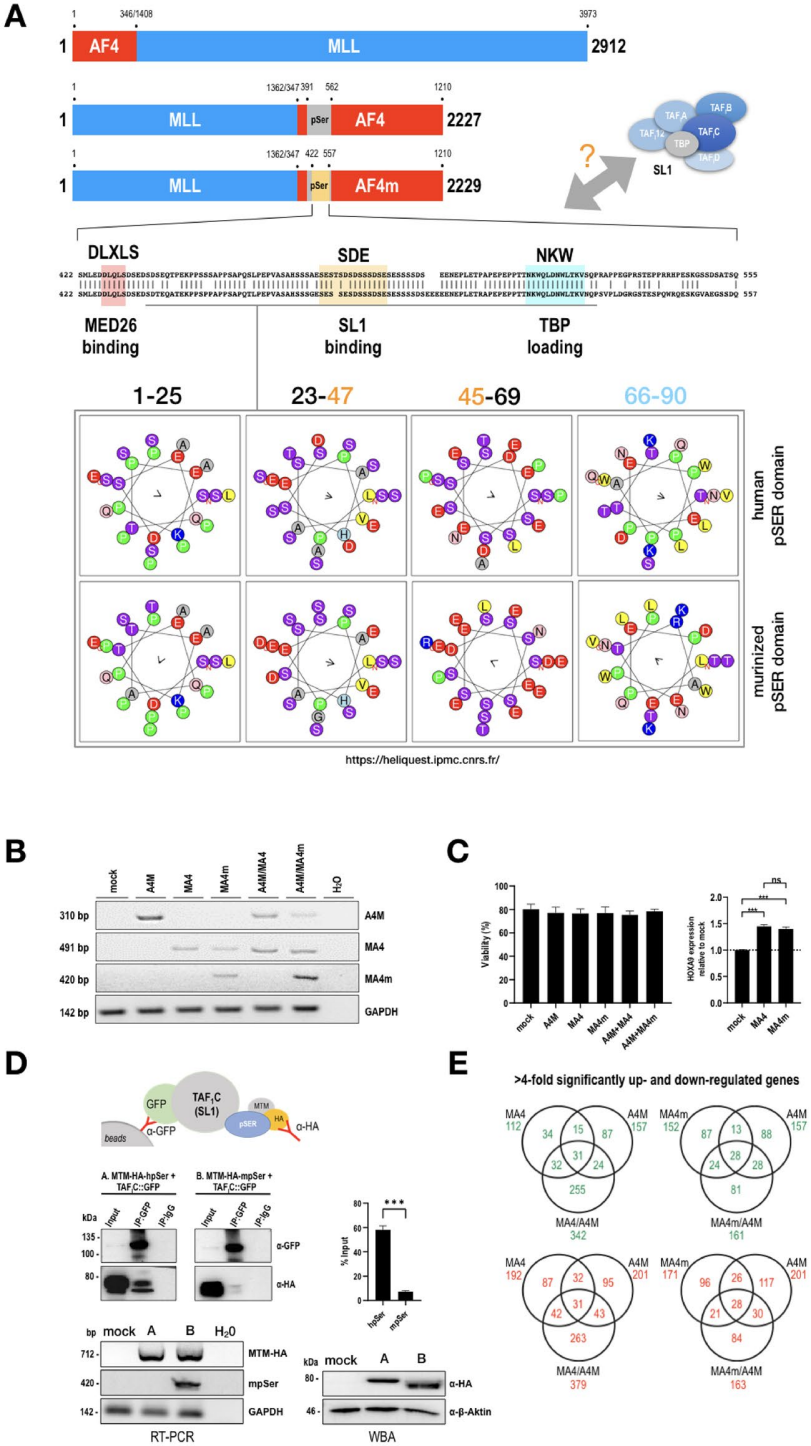
Design of and functional studies with the expression constructs MA4, MA4m and A4M. **A** Design of the A4M, MA4 and MA4m transgenes. Amino acids coordinates of fused portions and the pSer domain are indicated. The exchanged pSer domain contained the binding sites for the Mediator complex, SL1 and TBP. The helical wheel presentation of a portion of this sequence is shown below. This allows to visualize the differences between both sequences and consequences of missing or additional amino acids. **B** RT-PCR analyses of all inducible transgenes. These experiments validated the correct expression of all stably transfected transgenes. Sizes of each amplimer are given to the left. A GAPDH primer set was used to validate that equal amounts of cellular RNA were used in all experiments. **C** Cell viability and target gene validation. Cell viability was tested in independent experiments (n = 3). A single downstream target genes, *HOXA9*, was tested to validate the functionality of both the MA4 and MA4m fusion gene constructs. **D** Co-immunoprecipitation validated the binding of SL1 to the human pSer domain, while the murinized version binds to a eightfold lesser extent. Below: analyses demonstrating equal transcription of both MTM-(m)pSer domains (RT-PCR), and equal expression by Western blot analysis (WBA) **E** MACE-Seq experiment revealed the synergism between MA4 and A4M. MA4m has lost this ability

We used already established MLL-AF4 (MA4: MLL ex 1–9::AF4 ex 4–20) and AF4-MLL (A4M: AF4 ex 1–3::MLL ex 11–37) as a starting point to design first the murinized version of MLL-AF4 [23]. In order to substitute the corresponding part of the pSER domain, we compared the two homologous sequences that contain the three motifs DLXLS, SDE and NKW (see Fig. 1A). Both sequences are highly homologous, however, the human and murine deviated slightly at certain positions, with additional 3 missing amino acids in the human sequence and 1 missing amino acid in the murine counterpart. Since the latter amino acid was localizing in the SDE motif, we assumed that SL1 binding could potentially be compromized. This is also reflected by the helical wheel presentation where a portion of the shown amino acid sequence (aa 432–522 of human pSer or aa 432–524 from the murine counterpart) was used to display also the structural differences between both sequences. Additional or missing amino acids are changing the displayed protein surface, and thus, may influence the binding of interacting protein complexes.

By using molecular techniques, we therefore substituted the human AF4 sequence coding for AF4 amino acid position 422–555 by the murine counterpart (aa 422–557) and named this construct MLL-AF4m (MA4m).

Next, we established 5 stable cell lines that express either the 3 fusion gene constructs alone, or in combination with AF4-MLL (MA4/A4M and MA4m/A4M). For subsequent experiments, we also constructed a mock control cell line that contained only an empty vector pSBtet-P without expressing a Luciferase gene cassette). After transgene induction, isolated RNA was used to validate correct transcription of all 3 transgenes (a 310 bp A4M PCR fragment; a 491 bp MA4 PCR fragment). One primer pair detected specifically only the murine pSer domain to reassure the differences between the different cell lines (a 420 bp murine pSer PCR fragment; see Fig. 1B). Cells were grown in media to analyze cell viability and growth properties. However, repeated experiments revealed no significant changes in all 6 established cell lines concerning viability and cell growth (Fig. 1C, left panel). In order to validate functionality of the MA4 and MA4m constructs, a known target gene of MLL-AF4 was tested. As shown in Fig. 1C (right panel), transcription of *HOXA9* could only slightly augmented in both cell lines. These small changes can be explained by the already high expression of *HOXA* genes in the HEK293 cells, which is in contrast to leukemia cells or cells deriving from the hematopoietic compartment.

### Murinized MLL-AF4 represents a partial loss-of-function variant when binding to SL1

As described by Okuda et al., 2016, the SDE subdomain of the AF4 pSer domain is responsible for binding the SL1 transcription factor complex. Since both SDE motifs differ slighty between the human and the murine counterpart (see above), we first performed an Co-IP experiment in cells that were stably transfected with either MTM-HA-hum-pSer or the MTM-HA-mur-pSer expression constructs, and transiently transfected with the TAF_I_C::GFP expression construct. RT-PCR and Western blot experiments demonstrated the validity of both stable cell lines with correct and equal expression of the tagged MTM-HA-(m)pSer expression constructs (Fig. 1D, both lower panels). Precipitation was carried out with anti-GFP beads, while the detection of precipitated protein was carried out with anti-HA antibodies. As shown in Fig. 1D, the stably expressed proteins (MTM-HA-hpSer and MTM-HA-mpSER) were expressed at similar levels (see also “input”), while the TAF_I_C::GFP protein was able to nearly quantitatively pull-down the MTM-HA-hum-pSer protein, indicative for its strong interaction with SL1. By contrast, TAF_I_C::GFP could only partially pulled-down with the murinized MTM-HA-mur-pSer protein, indicating for an important difference between both MLL-AF4(m) fusions, namely a near loss-of-function situation with regard to SL1 binding capacity. The difference was about eightfold as shown in the quantification plot (Fig. 1D, right panel).

### Murinized MLL-AF4m is unable to synergize with the AF4-MLL fusion protein

We also performed MACE-Seq studies with all 5 cell lines, and used a mock cell line for normalization of the resulting data. As shortly summarized in Fig. 1E, short term expression of all transgenes (48 h) is leading to changes in gene transcription (only log2 >  ± 2 is shown in the presented VENN diagrams). As already published in a comprehensive study about the function of both reciprocal fusion proteins MA4 and A4M [23], here also a strong synergistic effect is visible when MA4 and A4M were co-expressed. This results in many deregulated genes (up- and down-regulated) in cells expressing both fusion proteins. This kind of synergy effect was gone when MA4m and A4M were coexpressed. This indicated for a different mode-of-action of both MLL-AF4(m) fusion protein which needed further investigations. These differences are also visible in Heatmap and Volcano plot analyses. Heatmap analysis shows that MA4 and A4M make synergistic actions and causes strong changes in gene transcription, while the combination of MA4m and A4M did not (Additional file 13: Fig. S1). Most signature genes in co-expressing cells mimics that one of MA4m. Similar results were observed in the volcano plot analysis (Additional file 13: Fig S2), where significant MLL (KMT2A) overexpression was visible in A4M expressing cells, while both co-expressing cell lines displayed AF4 (AFF1). Since MACE-Seq is quantitatively amplifying the 3′-end of RNA, this was an expected result. Noteworthy, AF4 was not visible in the single transfected cell lines expressing MA4 and MAF4, respectively. This demonstrates that the expression of these 2 fusion proteins is significantly lower in the absence of A4M expression, at least in case of both human fusion proteins. We also investigated the common and idiosyncratic protein coding gene sets (Additional file 13: Fig. S3 and S4) between MLL-AF4/MLL-AF4m (log2 >  ± 1) and the 2 cell lines co-expressing both fusion proteins (CO/COm cells; log2 >  ± 2). Also here, the amount of commonly deregulated genes is much lower than the idiosyncratic gene sets which are either activated (top) or downregulated (bottom). From the displayed gene sets it became clear that both fusion proteins have a different spectrum of target genes. All displayed genes are exclusively found only in the depicted cell lines (absent in the other 3 cell lines). All genes are listed from highest to lowest deregulated gene, while the number of reads, log2- and p-values can be retrieved from the accompanying Additional files 1, 2, 3, 4, 5, 6, 7, 8, 9, 10, 11, 12. Based on these gene expression studies, quite important differences were seen by changing the human pSer domain into the murine one.

### MA4 and MA4m both localize to the nucleolus and nucleus, but only MA4 redirects RNA Pol II from the nucleus to the nucleolus

**Fig. 2:**
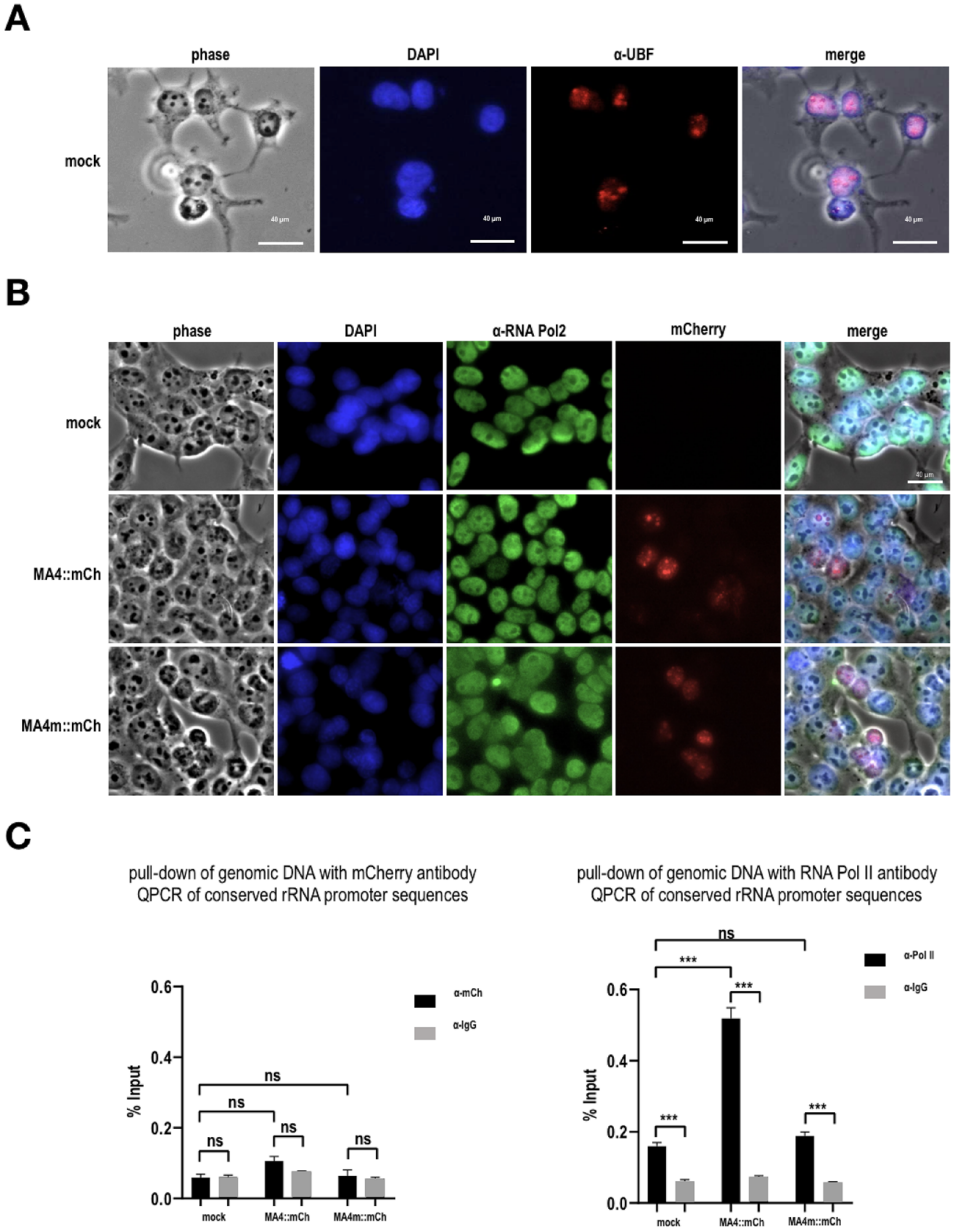
Overexpression of mCherry-tagged versions of MA4 and MA4m, and their intracellular localization. **A** Series of pictures taken with a fluorescence microscope showing the intracellular localization of UBF, a transcription factor necessary for RNA Pol I transcription inside the nucleolus. **B** Distribution of fusion genes and RNA Pol II in mock cells, as well as cells overexpressing the MA4::mCh and MA4m::mCh fusion proteins. Intracellular localization of both fusion proteins was observed in the nucleus, but also in nucleous (stronger in MA4 than in MA4m cells). **C** ChIP Experiments of genomic DNA performed with α-mCherry antibody and an α-RNA Pol II antibody. The two fusion proteins were unable to precipitate the investigated rRNA gene promoters, but the experiment with the α-RNA Pol II antibody revealed RNA Pol II binding to the investigated rRNA promoter when MA4::mCh was overexpressed

Since both constructs, MA4 and MA4m, were differently interacting with SL1 we got interested into the subnuclear localization of both fusion proteins. Therefore, we cloned an in-frame mCherry-Tag to the C-terminal portions of both MA4 and MA4m vector constructs (MA4::mCh or MA4m::mCh). Both constructs were again stably transfected and transgene expression was induced. Subsequently, the intracellular localization of both fusion proteins was investigated. As shown in Fig. 2A, mock-transfected cells were stained by an antibody against the important UBF factor which is necessary for RNA Pol I transcription. As shown in these experiments, UBF localizes precisely to the nucleoli of the investigated cells. Next, we analyzed the distribution of MA4::mCh and MA4m::mCh fusion fusion proteins. As shown in Fig. 2B, MA4::mCh seems to strongly stain nucleoli and slightly weaker the nucleus, while MA4m::mCh displayed a less intense nucleolar and nuclear staining. We also realized that RNA Pol II is less intense visible in cells that express MA4::mCh, while this was not the case in MA4m::mCh transfected cells.

Next, we investigated the ability of both fusion proteins, MA4 and MA4m, to bind directly to Pol I promotors, however, we could not see any direct binding of both tested fusion proteins to such promotor structures when analyzing the mCherry-pulled-down genomic DNA in Q-PCR experiments (Fig. 2C, left panel). Surprisingly, POL II was pulled-down with genomic DNA that represent a single conserved rRNA promoter (Fig. 2C, right panel, but only in MA4::mCh-transfected cells. These data may indicate that the observed accumulation of MA4::mCh in the nucleolus also resulted in a re-localization of RNA Pol II from the nucleus to the nucleolus. Thus, relocalized RNA Pol II co-immunoprecipitated with an rRNA promoter sequence that is usually bound by RNA Pol I. By contrast, the MA4m::mCh fusion protein had no such capacity, and is therefore less poisoness when expressed in mammalian cells.

### Changes in the morphology of nucleoli and rRNA promoter activity

**Fig. 3:**
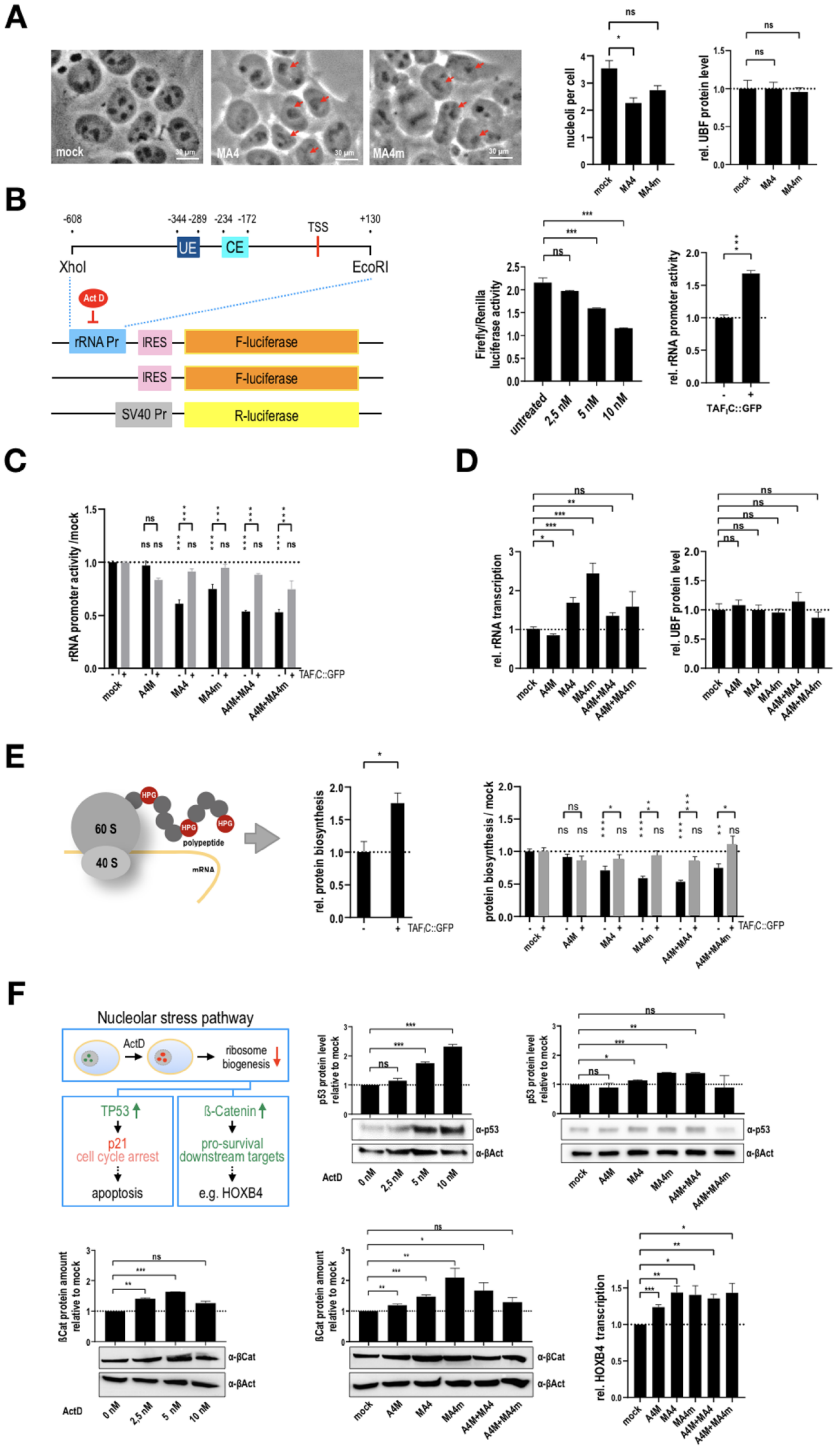
Nucleolus, rRNA synthesis and protein biosynthesis. **A** Morphology of nucleoli after transfection with MA4 and MAF4m. The morphology and number of nucleoli is grossly changed in the presence of both fusion proteins, MA4 and MA4m, respectively. **B** Design of the RNA Pol I Luciferase reporter gene. A single rRNA promoter has been used to set up a RNA Pol I dependent Luciferase reporter system that is responsive to Actinomycin D. **C** Reporter gene assays to monitor the activity of a single rRNA promoter in the presence of all tested fusion proteins or their combinations. The TAFIC::GFP fusion was able to enhance the rRNA promoter activity of the reporter gene. All fusion proteins caused a reduction of reporter gene activity. **D** QRT-PCR experiments of the total production of 45S precursors rRNA. Apart from A4M-single transfected cells, all other cell lines displayed an increasing amount of the 45S precursor RNA. The amount of UBF protein was quantified and remained stable in all tested cell lines. **E** Protein biosynthesis measured by the Click iT protein synthesis assay. Apart from A4M-single transfected cells, all other cell lines displayed a decreased protein biosynthesis rate. The reduced protein synthesis level could be rescued by the overexpression of TAF1C. **F** Investigating the nucleolar stress pathway. Either ActD or the presence of t(4;11) fusion proteins caused the steady-state upregulation of p53 and ß-Catenin. A downstream target of the WNT/ß-Catenin signaling pathway, *HOXB4*, was significantly upregulated by all tested fusion proteins

High resolution microscopy was used to study the structure and amount of nucleoli in the MA4- and MA4m-transfected cell lines. As shown in Fig. 3A, the morphology of nucleoli is changing when either of these both fusion proteins was expressed. We therefore first addressed the relative protein level of UBF which did not change significantly when nucleoli were stained by an anti-UBF antibody. However, the number of nucleoli became reduced significantly when MA4 is expressed (see Fig. 3A, right panels). There was a similar trend for a lower number of nucleoli in MA4m-transfected cells, however, this trend was not statistically significant.

The usual highly condensed structure of nucleoli became larger and was smoothened. Therefore, we asked the question whether RNA Pol I mediated transcription of the 45S precursor becomes affected. For this purpose, we developed a Pol I-dependent Luciferase reporter system (see Fig. 3B). This reporter system is based on a single rRNA gene promotor fragment (744 bp) that was cloned in front of an IRES and a Firefly Luciferase reporter gene. A construct without the RNA Pol I promotor fragment was used as negative control, and an SV40 promoter with Renilla Luciferase served as internal normalization vector for transfection experiments. The reporter system was functionally tested in a proof-of-principle experiment, namely by adding 2.5–10 nM Actinomycin D (ActD), a known inhibitor of RNA Pol I transcription (Fig. 3B, right panel 1). Vice versa, overexpression of a transiently transfected TAF_I_C::GFP led to an increase of rRNA promoter activity (see Fig. 3B, right panel 2). This validate the principal functionality of the reporter gene assay.

Subsequently, we transiently transfected into the 5 established cell lines and the mock-control cell line the newly established rRNA promoter reporter construct, and as an internal control additionally the TAF_I_C::GFP expression construct. This experiment revealed that MA4 cells—and to a lesser extend MA4m—caused a significant reduction of Luciferase activity which could be partially reversed by expressing additional TAF_I_C::GFP (see Fig. 3C). Luciferase activity was even further decreased in the presence of co-expressed A4M. We have to mention that the reporter plasmid is probably only present in nucleus of the transiently transfected cells, and thus, an interaction with RNA Pol I and TAF_I_C represents a limiting factor. Another concern was that the reporter plasmids may compete with endogenous rRNA gene transcription. Therefore, we needed to investigate also the production of 45S precursor rRNA in Q-PCR experiments (Fig. 3D, left panel) to understand the effects in more detail. As shown in this Figure, the endogenous steady-state amount of 45S rRNA was increased when either MA4 or MA4m was overexpressed. This increase was not so strong in the co-expressing cells. This controversial result could be explained by the fact, that we have cloned only a single rRNA promoter in our reporter plasmid, but there are many rRNA promoters (> 400) that differ slightly in their primary sequences and exhibit slightly different transcriptional activities. Since both fusion protein are unable to bind directly to rRNA promoter sequences (see Fig. 2C), and only MA4 was able to re-localize RNA Pol II to rRNA promoters, the lower increase of 45S RNA synthesis in the presence of MA4 may be explained by a competitive effect (RNA Pol II vs. RNA Pol I). Of interest, the amount of the UBF protein level remained unchanged when nucleoli were stained by an anti-UBF antibody and subsequently quantitatively analyzed (see Fig. 3D, right panel). This demonstrated that the total number of rRNA gene copies (usually ~ 200 copies out of the > 400 copies) used for 45S rRNA precursor transcription remained unchanged [24]. Thus, it could be that the presence of MA4 or MA4m are probably only changing the architecture of the nucleoli, and thus, are influencing indirectly which rRNA genes are being transcribed. Depending on which promoter regions are being transcribed by RNA Pol I, or by changing the morphology of the nucleolar structure, this influenced positively the amount of transcribed 45S precursor rRNA.

### Influence of MA4 and MA4m on protein biosynthesis

Having seen that more 45S rRNA is being produced in the presence of MA4 or MA4m, we were interested in finding out the direct effect on protein biosynthesis. By using the commercially available Click-it protein synthesis assay (Thermo Fischer Scientific), which measures quantitatively the production of proteins at ribosomes, we were able to demonstrate that stably transfected cells expressing only MA4 or MA4m displayed a significantly reduced protein biosynthesis (see Fig. 3E). Co-expression of MA4 and A4M had the strongest effect, while the combination of MA4m and A4M seem to cause less inhibition of protein biosynthesis (no synergism). These negative effects on protein biosynthesis could be partially reversed by the additional over-expression of TAF_I_C::GFP. In summary, nucleolar stress is playing a major role when the fusion proteins MA4 or MA4m are expressed, and the increased transcription of 45S precursor rRNA shown in Fig. 3D could simply reflect for a compensatory mechanism in order to cope with this nucleolar stress phenomenon. However, if the presence of fusion proteins interfer with cellular protein synthesis, then MA4m or MA4/A4M had the strongest effect, while co-expression of MA4 and A4M the least effect. Thus, the presence of A4M ameliorates the negative effect of MA4, but not that one deriving from MA4m.

### MA4 and MA4m are both turning on the nucleolar stress pathway which results in the stabilization of the ß-catenin protein

In order to understand the molecular consequences of impaired protein biosynthesis, we investigated the role of “nucleolar stress” [25] in the presence of these fusion proteins. As shortly summarized in Fig. 3F, the nucleolar stress pathway turns on when cells are unable to produce enough ribosomes to cope with the cellular requirements of protein biosynthesis. Supernumerary ribosomal proteins not being involved in ribosome subunit production are able to interact with nuclear MDM2, which usually binds and triggers the proteasomal degradation of p53. Therefore, during a “nucleolar stress response” a slightt increase of p53 is usually observed, which in turn causes a p21-dependent cell cycle arrest and apoptosis. In addition, inhibited production of 45S RNA—or its subsequent processing into 28S, 18S and 58S rRNA (by PPAN, NPM, PES1 or SBDS)—is leading to an GSK3ß-independent accumulation of cytosolic ß-Catenin, which in turn causes the artificial transcriptional upregulation of WNT/ß-Catenin target genes in order to bypass the nucleolar stress conditions [25].

To validate this assumption in case of MA4 or MA4m expression, we performed a series of experiment to get a first hint that this nucleolar stress pathway is turned on. In fact, a simple treatment with Actinomycin D in various concentrations (2.5–10 nM) led to the accumulation of p53 and ß-Catenin (see Fig. 3F, middle upper and lower left panel). When analyzing the 5 cell lines together with the mock control, p53 was slightly induced either by MA4m alone, or, by the combination of MA4 and A4M. Of interest, this nucleolar stress phenomenon was fading away when MA4 was co-expressed with A4M (see Fig. 3F, upper right panel), demonstrating again that A4M has an important function to ameliorate negative effects deriving from the MA4 fusion protein.

In addition, an increase of ß-Catenin was observed strongest with MA4m alone, followed by the combination of MA4/A4M and MA4 alone (see Fig. 3F, lower middle panel). A common pro-survival WNT/ß-Catenin target gene of this nucleolar stress pathway is *HOXB4*, which was significantly induced in all 5 cell lines expressing the various combinations of t(4;11) fusion proteins, indicating that the presence of these fusion proteins—including the artificial MA4m fusion construct—are all triggering the nucleolar stress response pathway, presumably to prevent p53-mediated apoptosis.

## Discussion

This paper aimed to understand the differences of MA4 and MA4m fusion proteins which differ only slightly in their pSer domain (either human or murine sequences). The motivation to perform such a study came from differences in experimental results made in different laboratories when working with t(4;11) fusion proteins, as well as the use of murine protein sequences to eliminate potential negative effects deriving from human AF4 protein sequences that seem to prevent ALL development [1, 13]. Another hint that stimulated our study came from studies on AF4 and SL1 [2], a transcription factor complex that is necessarily involved in the transcription of 45S precursor rRNA in mammalian cells [18–22]. SL1 is composed by TAF_I_12, TAF_I_A, TAF_I_B, TAF_I_C, TAF_I_D and TBP. Of note, TAF_I_C has already been shown to exhibit the strongest binding to the pSER domain which made it attractive to use it as experimental read-out system. These studies, which dissected the process of SL1 binding to specific subdomains of the AF4 pSer domain, gave the motivation to investigate this in the context of full-length fusion protein, in order to understand the biological consequences in changing human and mouse protein sequences, which should shed some new light onto the enigmatic history of the MLL-AF4 fusion protein deriving from balanced t(4;11) chromosomal translocations [17, 27]. The pSer domain of AF4 is always fused to the N-terminal portion of MLL in all yet investigated patients with a t(4;11) translocations, regardless of the breakpoint localization within the *MLL* gene [3]. It contains the three subdomains DLXLS, SDE and NKW, respectively (see Fig. 1A). SDE and NKW were shown to be important for transactivation and transforming ability, with an SDE subdomain to be important as SL1-binding platform, while the NKW subdomain is needed to initiate RNA Pol II-dependent transcription.

Since Lin et al. used the complete *Af4* sequence in their hMLL::mAf4 construct to express the MLL-Af4 fusion protein, we decided to investigate only the SL1 binding site of AF4 or Af4 as potential explanation for their observed differences in leukemia onset and development [1].

Our first attempt to compare the pSer domain between both orthologous protein sequences already identified some minor differences in the primary sequence and potential surface that could be tested in our experimental setting. As shown in Fig. 1A, we substituted a portion of the AF4 pSer domain (134 amino acids) within the MLL-AF4 (MA4) by the orthologous mouse sequences (136 amino acids). This novel construct was termed MLL-AF4murine (MA4m). Next, we used our established Sleeping Beauty technology [28] to generate a series of stably transfected cell lines that allowed us to investigate potential differences in MA4- and MA4m-transfected cell lines, or to test both direct MLL fusion proteins in conjunction with the reciprocal AF4-MLL fusion protein (A4M).

The first important finding was made when we investigated the binding of the SL1 complex to both pSer domains. We used not the full-length MA4(m) constructs, but a mini-MLL-AF4 version designed in Akihiko Yokoyama's lab [2]. By using a GFP-tagged version of TAF_I_C, we could precipitate these mini-MLL-pSer domain fusion proteins. However, while the human mini-MLLpSer was strongly precipitated, the murine counterpart did not effectively bind to TAF_I_C (see Fig. 1D). The quantification revealed that murine pSER binds ~ 8-times less efficient than the human pSer domain. Both domains can be distinguished by a single missing amino acid (SDE: SES-(T)-SDSDSSSDSE), but the surface of the folded proteins may be more important, as dramatic changes may occur when single amino acids are missing or were added (see helical wheel visualization of this protein portion in Fig. 1A).

When we analyzed the intracellular localization of mCh-tagged full-length fusion proteins, MA4:.mCh and MA4m::mCh, we had another surprising result: although we know that MLL-AF4 fusion proteins are distributed in the cell nuclei and binding to their specific target genes, the majority of these fusion protein was localizing in the nucleolus (Fig. 2A). This was true for MA4::mCh, and to a lesser extent for MA4m::mCh. This indicated that a major fraction of the overexpressed fusion proteins is entering the nucleolus and potentially interfering with 45S precursor rRNA synthesis or subsequent ribosome biosynthesis. A minor fraction of these fusion proteins were still present inside the nucleus and able to activate their specific target gene (see Additional file 13: Fig. S3 and S4, as well as Additional file 1–12: Excel files). Noteworthy, more available MA4m fusion protein in the nucleus will allow a more effective binding to target genes (Additional file 13: Fig. S3) which could be an explanation for the finding of Lin et al. [1]. MA4m alone could upregulate more protein coding genes than MA4A alone. Interestingly, MA4 alone downregulated even genes like e.g. MEIS3 and RUNX1. This pictures was changing when A4M was co-expressed with either of these direct fusion proteins, and the number of target genes (up- or down-regulated was strongly increased only when both reciprocal fusion proteins were completely human (see Additional file 13: Fig. S4). Another effect caused by these fusion proteins was that RNA Pol II was able to enter the nucleolus in the presence of MA4 (strong) and MA4m (slightly weaker). RNA polymerase II was even shown to interact somehow with rRNA promoter sequences in the presence of MA4 (Fig. 2C). This means that RNA Pol II is recruited away from the nucleus to the nucleolus, and thus, normal gene transcription may become a limiting factor in the absence of the reciprocal A4M. Of note, A4M was recently shown to strongly increase gene transcription processes by playing the role of a chromatin opener [15, 23].

Also in this study, we investigated target gene transcription by performing MACE-Seq experiment. By using biological triplicates we investigated the target gene spectrum of MA4 alone, MA4m alone, A4M alone and the 2 co-expressing cell lines. In the VENN diagrams shown in Fig. 1E, the up- and down-regulated target genes in MA4 (112 up- and 192 down-regulated genes), A4M (157 up- and 201 down-regulated genes) and MA4/A4M (342 up- and 379 down-regulated genes) clearly pointed to a synergistic activity when both reciprocal fusion protein are expressed. The idiosyncratic target gene spectrum with MA4m alone was only slightly higher for the upregulated target genes (152 up- and 171 down-regulated genes), but any synergistic cooperation with A4M was lost (161 up- and 163 down-regulated genes). A more detailed analysis is shown in Additional file 13: Figs. S3 and S4, as well as in the accompanying Excel files, however, we were explicitely not interested here in target gene analysis rather in the differences between MA4 and MA4m and the ability of A4M to enhance or counteract features deriving from both fusion proteins.

Next, we investigated the morphology of nucleoli in more detail and could see that their compact and condensed structure becomes less defined and more smoothened in the presence of MA4 and MA4m, respectively (see Fig. 3A). Despite these changes in morphology, also the number of nucleoli was slightly reduced, while the amount of the UBF protein, important for binding to all active rRNA gene promoters, remained constant in all investigated cells (Fig. 3A, right panel). To this end, both fusion proteins in conjunction with presence or absence of RNA Pol II seem to change the architecture of nuceoli. How this interfers with normal nucleolar functions remained to be investigated.

To answer this important question we have set up a novel tool to investigate rRNA promoter activity in a quantitative fashion. One of the many repetitive rRNA promoters was cloned to set up a new reporter system with which we aimed to investigate Pol I activity. The cloned promoter element contained the necessary binding sites for UBF (upstream element UE and core element) and SL1 (core element CE) as well as the transcriptional start site (TSS; see Fig. 3B). By using this reporter construct (together with a negative and internal control), we could show that the addition of up to 10 nM Actinomycin D—a poison for RNA Pol I transcription—functionally impaired the reporter gene activity. By contrast, the transient overexpression of a TAF_I_C::GFP fusion protein increased the relative rRNA promoter activity (see Fig. 3C).

Subsequently, we transiently transfected the reporter plasmids (rRNA Promotor-IRES-LUC or IRES-LUC ± TAF_I_C plasmid) into all 5 stable cells lines. Unfortunately, we revealed that all fusion proteins—with the exception of A4M—displayed a much lower Luciferase activity (see Fig. 3C). The co-expression of TAF_I_C::GFP reverted the observed inhibitory effects. In addition, we also analyzed the total 45S precursor rRNA to get insight into potential stress effects. As shown in Fig. 3D, we observed elevated levels of 45S precursor in the presence of MA4 and MA4m, while the presence of additional A4M lowered the observed effects for both reciprocal constructs. This observation may in part be explained by the fact, that both MA4 and MA4m fusion proteins are localizing to the nucleolus, and thus, available SL1 complex in the nucleus may become a limiting factor for Pol II transcription in the nucleus. Since MA4m is less localizing to the nucleolus and less binding to SL1, this particular fusion protein is displaying less inhibitory functions on the rRNA promoter reporter assay.

The only critical point in our experimental results is the comparison of 45S rRNA precursor production (Fig. 3D) and the ability to produce protein (Fig. 3E). We have seen a higher production of precursor rRNA, but a reduced capability of protein biosynthesis (Fig. 3E). There could be many reasons (e.g. a disturbed maturation process, a disturbed subunit production, a disturbed export function, a disturbed mRNA production, etc.). All this has not been investigated here, but the expression of t(4;11) fusion proteins—except for A4M—is somehow interfering with this important pathway. Any disturbance on either rRNA synthesis or subsequent pathways has usually dramatic effects for cells. It is known that cells do not tolerate changes in this "ribosome biosynthesis system" and are quite sensible to even tiny changes. Overexpression of TAF_I_C was able to partially compensate these reductions, which again points to the importance of the SL1 complex for this pathway. We also looked to the literature to find an explanations for the higher production of the 45S precursor in the presence of t(4;11) fusion proteins. An interesting observation has been made recently when studying ribosome biosynthesis [29]. RNA Pol II is quite important in nucleoli for the biosynthesis of rRNA, because RNA Pol II—associated with Senataxin (*SETX*)—transcribes intergenic regions between rRNA genes, and thus, inhibits the formation of sense intergenic noncoding RNA (sincRNA) produced by RNA Pol I. This kind of "shielding effect" is caused by R-loop formation during transcription of RNA Pol II which in turn allows to maintain high yields in rRNA production. Thus, RNA Pol II inside of nucleoli has a quite important biological function which seems to be enhanced by the presence of MA4, but not by MA4m. The MA4 fusion protein seems to increase the relocalization of RNA Pol II, however, not exerting the recently described shielding effects, because we observed RNA Pol II at RNA Pol I promoters and a decreased protein biosynthesis rate.

The molecular consequences of the “nucleolar stress pathway” is depicted in Fig. 3F. Usually, nucleolar stress is translated molecularly into the stabilization of p53, which would result in cell cycle arrest and apoptosis. However, cancer cells need to divide, and thus, this part of the pathway would be rather contraproductive than helpful. Therefore, a second pathway is linked to the nucleolar stress pathway, which is the stabilization of ß-Catenin, bypassing the phosphorylation of GSK3ß (inactivation) and counteracting the adverse effects of p53 by activating pro-survival target genes. One of the known downstream target genes is the pro-survival gene *HOXB4* [26]. We could show in our Western blot experiments with Actinomycin D (as an pharmacological inductor of the nucleolar stress pathway) or by the expression of the t(4;11) fusion proteins that we could show a slight increase of p53 protein abundance, but moreover a strong increase of the ß-Catenin protein. In addition, this increased ß-Catenin levels translated directly into a higher transcription rate of *HOXB4* in QRT-PCR experiments. To this end, MA4, MA4/A4M but mostly MA4m caused an increase of steady-state ß-Catenin levels, while the combination of MA4m and A4M did it not significantly, although a transcriptional *HOXB4* activation was even seen under these conditions.

Probably t(4;11) cells may use the nucleolar stress pathway to enhance ß-Catenin signaling without involving GSK3ß. ß-Catenin is known as direct binding partner of GSK3ß as part of the canonical WNT signaling pathway. ß-Catenin has already been identified as a key molecule in *MLL*-r acute leukemias by causing an increasing self-renewal and proliferation of LIC's [30, 31]. As an example, ß-Catenin was show to cooperate with MLL-AF9 in order to develop into aggressive leukemia, while ß-Catenin k.o. mice were quite restricted to develop leukemia in the presence of MLL-AF9, and finally, a pharmacological depletion of ß-Catenin by Indomethacin had the similar effect of preventing leukemia development [30]. An elevated ß-Catenin level also reduces the sensitivity of GSK3ß to GSK3ß inhibitors, which in turn lead to a resistance of leukemias against such a therapy [31]. This effects might be even super-enhanced by FRAT1 and FRAT1 which are overexpressed target genes in in *MLL*-r leukemias and allow to disable GSK3ß, which in turn increase again the steady-state ß-Catenin protein levels and cause subsequntly an increased self-renewal and proliferation capacity [32]. The FRAT/ß-Catenin connection also result an increased RAC/RHO signaling which also contributes to self-renewal and survival. To this end, an increase of endogenous ß-Catenin (e.g. by the nucleolar stress pathway) is functional equivalent to an inhibition of GSK3ß. This way, cells become probably independent from external signals without losing their self-renewal or proliferation capacity.

## Conclusions

Several questions are still remaining, however, this manuscript shed some fresh light into the mechanisms that were executed in the presence of t(4;11) fusion proteins. Here, we present first evidence that the MLL-AF4 fusion protein—or a variant thereof—is influencing one of the most sensible pathways in our cells, namely processes in the nucleolus and protein biosynthesis.We identified that the t(4;11)-derived MLL-AF4 fusion protein displays [1] cooperativity with the reciprocal AF4-MLL fusion protein, [2] recruits and binds strongly to the SL1 transcription factor complex, [3] targets and deregulates nuclear gene transcription, and [4] localizes to and recruits RNA Pol II to the nucleolus and [5] interfers with the 45S precursor rRNA production and protein biosynthesis. Exchanging the pSer domain within the MLL-AF4 fusion protein by the homologous murine sequences (~ 130 amino acids) nearly [6] nearly abolishes the recruitment of SL1 to MLL-AF4m which [7] localizes now more in the nucleus than to the nucleolus, where it [8] deregulates a different set of target genes. These data may explain in part the differences in the leukemogenic behavior of MLL-AF4 (not leukemogenic) and hMLL-mAF4 (which seem to be leukemogenic) at the molecular level. Moreover, this study provides also a new twist into an important mechanism that results in increased levels of ß-Catenin, which was already shown in the past to be essential for leukemic initiating cells in other *MLL*-r leukemia systems.

## Methods and materials

### Cell culture and transfections

HEK293T cells were grown in DMEM with 10% (v/v) FCS (Capricon Scientific), 2 mM L-Glutamine (Capricon Scientific), and 1% (v/v) Pen Strep (GE Healthcare) at 37 °C and 5% CO_2._ Stable cell lines were established using an optimized Sleeping Beauty Transposon System [28]. 50 ng of SB transposase vector SB100X and 1 µg of the respective plasmid(s) were applied with Metafectene Pro® (Biontex) (pSBtet::MLL-AF4, pSBtet::MLL-AF4m, pSBtet::AF4-MLL, pSBtet::TAF_I_C::GFP). After 24 h, cells were subjected to either Puromycin (AF4-MLL, 2 µg/ml) or Blasticidine (MLL-AF4, MLL-AF4m; 15 µg/ml). The cells were incubated with selection markers for 3–10 days. Transgene induction was carried for at least 48 h with 1 µg/ml Doxycycline. Another cell line was created by stable transfection of the pSBtet-P vector (without Luciferase) and used throughout the experiments as mock control.

### Plasmid constructions

The plasmid encoding the MTM-HA-hum-pSer gene was kindly provided by Akihiko Yokoyama (Tokyo, Japan; 2), and was used to replace to human pSer domain by the murine counterpart to obtain the MTM-HA-mur-pSer construct. Both constructs were cloned via *Sfi*1 sites into pSBtet-P.

The TAF_I_C::GFP construct was designed by fusing the open reading frames of TAF_I_C with that one of super-folder GFP (pET29BH4:10xHis-TEV-sGFP was a gift from Dr. Jan Hering, Frankfurt, Germany) to obtain the final constructs. This construct was cloned via *Sfi*1 sites into pSBtet-B. TAF_I_C is central part of SL1 and was already shown to bind strongest to the pSer domain of AF4 [1], and thus, was used in all experiments to represent SL1 binding to MA4 or MA4m.

The MA4::mCh and MA4m::mCh constructs were designed by eliminating the terminal stop codon and fusing the open reading frame to the mCherry open reading frame. The two final constructs were cloned into pSBtet-P.

All cloned transgenes were induced by adding 1 µg/ml Doxycycline for exactly 48 h before any experiment was performed. Based on our experience with Sleeping Beauty vector systems, this is the best timepoint for full expression of transgenes.

The rRNA promoter sequence was cloned by PCR from the human genome with the 2 oligonucleotides pHrRNA.F (5′-CACCTCGAGCGCGATCCTTTCTGGAGAGTCCC-3′) and pHrRNA.R (5′-AAGCGAATTCGACGAGAACGCCTGACACGCAC-3′), digested with *Xho*I and *Eco*RI and cloned as a 754 bp long DNA fragment into the pGL3-IRES-Basic (Addgene) to obtain a Pol I Luciferase reporter plasmid. pGL3-IRES served as negative control. A SV40-Renilla Luciferase construct served as internal standard for all experiments. The ribosomal promoter element contained the upstream element (binding site for UBF) and the core element (binding site for UBF and SL1), as well as the transcriptional start site.

### RNA extraction, cDNA synthesis and RT-PCR experiments

In all 6 stable cell lines, transgene induction was carried by using 1 µg/ml Doxycycline for 48 h. Total RNA was isolated by using RNeasy® Mini Kit (Qiagen) and cDNA synthesis were performed using SuperScript® II (Invitrogen). All isolated RNAs were quality checked (Agilent Bioanalyzer) and final concentrations were determined. Equal amounts of total RNA were used throughout all experiments, and all experiments were performed with 3 biological replicates. Primers used for RT-PCR analyses are as follows: A4M.F 5′-TCCGGCCCATGGATGGTCAAGATCAGGC-3′, A4M.R 5′-TTGTGGAAGGGCTCACAACAGACTTGGC-3′, MA4.F 5′-ACCTACCCCATCAGCAAGAGAGGATCCTGC-3′, MA4.R 5′-GCCATGAATGGGTCATTTCCTTCAGAATCT-3′, Af4.pSer.F 5′-CGTCTCCATGCTGGAGGACGACCTGCAGCTCAG-3′ and Af4.pSer.R 5’AGAATGCTCCTGGTCACTGCTGCCCTCAGCGACA-3′. Target gene transcription was quantified in Q-PCR experiments with the following primers: HOXA9.F (5′-CAATGCTGAGAATGAGAGCGG-3′), HOXA9.R (5′-TGTATAGGGGCACCGCTTTTT-3′); HOXB4_RT.F (5′-CCTGGATGCGCAAAGTTCAC-3′), HOXB4_RT.R (5′-CCTTCTCCAGCTCCAAGACC-3′), GAPDH.F 5′-GGTCACCAGGGCTGCTTTTA-3′, GAPDH.R 5′-CGTTCTCAGCCTTGACGGTG-3′, qPCR_45SrRNA_28S.F (5′-CGATCTATTGAAAGTCAGCCCTCGACACAAGG-3′) and qPCR_45SrRNA_3′ETS.R (5′-CGGTCGGCGGGAGAGGCCGGGAGGGAGGAAGACGAACG-3′).

### Differential gene expression profiling by MACE-Seq

For the MACE-Seq experiments, all cell lines were treated with 1 µg/ml Doxycycline for 48 h with and total RNA were isolated from transfected cell lines. After testing the correct expression of transgenes, differential gene expression (DGE) profiles were obtained by MACE (Massive Analysis of cDNA Ends)—Seq experiments following the manufacturer protocol (GenXPro, Frankfurt, Germany). Three biological replicates of each cell line were compared with 3 biological replicates of mock-transfected cells. The MACE-libraries were prepared at GenXPro GmbH using the Massive Analysis of cDNA Ends (MACE) Library Preparation Kit (v2.0) from GenXPro GmbH. First, cDNA was generated using Oligo(dT) primers with distinct Oligo IDs per sample for subsequent pooling of up to 24 samples. After pooling, cDNA was fragmentated to an average size of 200 bp using the sonicator Biorupter Plus (Diagenode, Belgium). The distribution of cDNA fragment sizes was monitored using the automated microfluidic electrophoresis station LabChip GXII Touch HT platform (PerkinElmer, USA). The Poly(A) containing cDNA fragments were purified using solid phase reversible immobilization (SPRI) beads (Agencourt AMPure XP, USA), end repaired and ligated to distinct 8-base pair UMI Adapters (also called TrueQuant adapters). Then, the library containing labelled and fragmentated cDNA was amplified by PCR, purified by SPRI beads (Agencourt AMPure XP, USA) and strand-specific sequenced using the HiSeq2500 (Illumina, USA).

Bioinformatic analysis was performed according to the analysis pipeline for MACE libraries by GenXPro GmbH. Unique Oligo IDs and UMIs on each transcript allowed initial demultiplexing and subsequent removal of PCR-duplicates. The remaining reads were trimmed for high-quality as well as adapter-free sequences and aligned to the human reference genome (Genome Reference Consortium Human Build 38 patch release 13 (GRCh38.p13) using Bowtie 2. Resulting output data were implemented in the database program FileMaker for further analysis. All data received from the Bioconductor software from the MACE-Seq experiments were incorporated into a FILEMAKER database program. In addition, we used the following server for further data analysis: Heatmapper (http://www.heatmapper.ca/expression/) for heatmap analyses and VolcaNoseR (https://huygens.science.uva.nl/VolcaNoseR/) for volcano plots.

### Antibodies used throughout this study

The following antibodies have been used throughout this study: anti-β-Catenin (Cell Signaling, #8480), anti HA-Peroxidase (Sigma Aldrich, #34071100), anti rabbit IgG-Peroxidase (Abcam, ab6721; secondary antibody, Western Blot), anti mouse IgG-Peroxidase (Abcam, ab97023; secondary antibody, Western Blot), anti GFP (Abcam, ab290), anti UBF (Santa Cruz, sc-13125), anti mCherry (Abcam, ab125096), anti β-Actin-Peroxidase (Sigma Aldrich, A3854), anti RNA Polymerase II(Diagenode, AC-055-100), anti mouse IgG-Alexa Fluor®586 (Abcam, ab175473; secondary antibody, IHC), anti p53 (Santa Cruz, sc-47698), Anti-RNA polymerase II CTD repeat YSPTSPS (phospho S2) (Abcam, ab5095), Goat Anti-Rabbit IgG H&L (FITC) (Abcam, ab6717) respectively.

### Cell fixation and immunofluorescence staining and detection

HEK 293 T cells lines were cultivated on Poly-D-Lysin pretreated glass chamber slides and transgene expression was induced for 48 h with 1 µg/ml Doxycycline. Next, cells were washed with PBS containing 1 mM CaCl_2_ and 0.5 mM MgCl_2_ and then fixed for 20 min in cell fixing solution (3.7% Formaldehyde (v/v) in PBS + 1 mM CaCl_2_, 0.5 mM MgCl_2_) following quenching in 50 mM Glycin in PBS + 1 mM CaCl_2_, 0.5 mM MgCl_2_ for 5 min. After repeated washing with PBS, cells were permeabilised for 15 min in a permeabilisation solution (0.2% Triton™ X-100, 0.1% SDS in PBS + 1 mM CaCl_2_, 0.5 mM MgCl_2_).

In case of immunostaining, glass slides with fixed cells on the surface were blocked in a Coplin Jar with TBST with 5% BSA for 1 h and afterwards incubated in TBST diluted primary antibody o/n at 4 °C (1:1000–1:8000). The next day cells were washed with TBST and incubated in TBST diluted secondary antibody for 1 h at RT (1:10.000). After repeated washing with TBST stained cells were embedded in Duolink® In Situ Mounting Medium with DAPI (Sigma Aldrich) and analysed with the fluorescence microscope Observer Z1 (Carl Zeiss).

For Quantification of UBF protein levels 1 × 10^4^ cells of each stable transfected HEK 293 T cell line were seeded in triplicates in a 96-well plate and incubated for 48 h with Doxycycline. After the fixation procedure as mentioned above, an antibody incubation was carried out in TBST o/n and for 1 h respectively. After washing with TBST, 50 μL of HCS NuclearMask™ Blue Stain (H10325, Thermo Fisher Scientific) was added per well and incubated 30 min at RT protected from light. Afterwards, all wells were washed again and 100 µl of TBST was added before measuring flourescense signals at the Varioskan Flash plate reader (Thermo Fisher Scientific). The analysis was performed by normalization to DAPI and mock.

### Q-PCR experiments

All quantitative PCR analyses were performed with the StepOnePlus™ System (Applied Biosystems). All measurements were normalized to the Ct values of *GAPDH* of mock transfected cells and were analyzed in triplicates. The results were evaluated by the comparative ΔΔCt method.

### Viabilitätsassay

For the determination of cell viability 1 × 10^6^ HEK 293 T cells were seeded into 10 cm cell culture dishes and transgene expression was induced for 48 h with 1 µg/ml Doxycycline. Cells were detached by Accutase® (Capricorn) treatment and an Aliquot was mixed with Acridine Orange and DAPI containing Solution (Chemometec) and analysed with the Nucleocounter NC-3000™ (Chemometec) according to manufactors instructions.

### Luciferase reporter assay

The rRNA promoter acitvity was measured using the Dual-Luciferase® Reporter Assay System from Promega. 4 × 10^5^ HEK 293 T cells were seeded in a 6-well plate in triplicates. The expression of transgenes was induced by the addition of 1 µg/ml Doxycycline for 48 h. 24 h prior to analysis, cells were transiently transfected with reporter and control vectors. Measurement of Luciferase activities was performed according to manufactors instructions.

### Western blot

5 × 10^5^ cells of each HEK 293 T cell line were cultivated in 6-well plates for 48 h with 1 µg/ml Doxycycline for induction of transgene expression. Afterwards cells were lysed for 45 min in 50 µl lysis buffer (1% Triton X-100 (v/v), 1% Deoxycholat (w/v), 1 × protease inhibitor cocktail (Roche) at 4 °C. Cell lysates were obtained after centrifugation at 13.000 rpm for 10 min. Whole cell lysate was loaded onto a 10% SDS Gel. Seperated proteins were transferred onto a PVDF membrane using the standard protocol for Trans-Blot TURBO system (BioRad). After blocking in TBST + 5% BSA for 1 h at RT, membranes were incubated in primary antibody o/n at 4 °C. The next day membranes were washed in TBST and incubated in secondary antibody for 1 h at RT following detection using the Clarity™ ECL Western substrate and Chemi DOC™XRS + Imager (Biorad).

### Co immunoprecipitation

1 × 10^7^ HEK 293 T cells were seeded into a 15 cm cell culture dish with 1 µg/ml Doxycycline for induction of transgene expression for 48 h. The Medium was discarded, cells were washed with ice cold PBS, resuspended in lysis buffer (150 mM NaCl, 10 mM Tris–HCl, 1 mM EDTA, 1 mM EGTA, 1% Triton X-100 (v/v), 0,5% NP40 (v/v), 1 × Protease Inhibitor Cocktail (Roche)) and incubated for 30 min on ice. After centrifugation at 1000 rpm for 5 min at 4 °C the cell lysate was transferred into a new 1.5 ml reaction tube. Protein concentration was determined with Pierce™ BCA Protein Assay (Thermo Fisher Scietific) and 200 µg protein was used per IP which was performed according to NEB protocol.

### Chromatin immunoprecipitation experiments

ChIP experiments were performed using the Abcam protocol. Stably transfected HEK 293 T cells (1 × 10^7^ cells on a 145-mm cell culture plate) were transgene-induced with 1 µg/ml Doxycycline for 48 h. For double fixation, the cells were incubated with 2 mM di(N-succinimidyl)glutarate for 45 min and 1% (v/v) formaldehyde for 10 min. Sheared chromatin was incubated with magnetic A/G beads and antibodies overnight following precipitation. Quantitative PCR analysis was performed with the percent input method from ThermoFisher Scientific by using the following primers: rRNA.Prom.for (5′-GGCTGCGATGGTGGCGTTTTTGG-3′) and rRNA.Prom.rev (5′-GGACAGCGTGTCAGCAATAACCCG-3′).

### Click iT protein synthesis assay

The analysis of protein biosynthesis in HEK 293 T celllines was performed with 2 × 10^4^ cells in each Poly-D-Lysin pretreated 96-well plates after 48 h induction with 1 µg/ml Docxcyclin with the “Click-iT™ HPG Alexa Fluor™ 594 Protein Synthesis Assay” (Invitrogen) according to manufactors instructions. Experiments were performed with 6 biological replicates per cell line.

## Supplementary Information

**Additional file 1: **MA4 gene signature.

**Additional file 2: **MA4m gene signature

**Additional file 3: **A4M gene signature.

**Additional file 4: **MA4_A4M gene signature.

**Additional file 5: **MA4m_A4M gene signature.

**Additional file 6: **Heatmap dataset 1.

**Additional file 7: **Heatmap dataset 2.

**Additional file 8: **MA4 volcano.

**Additional file 9: **MA4m volcano.

**Additional file 10: **A4M volcano.

**Additional file 11: **MA4_A4M volcano.

**Additional file 12: **MA4m_A4M volcano.

**Additional file 13: Figure S1.** Bioinformatic analysis of MACE-Seq data-I Heatmaps analysis. **Figure S2.** Bioinformatic analysis of MACE-Seq data-II Volcano plot analysis. **Figure S3.** Investigation of protein coding genes which are common and unique for MA4 and MA4m cells. **Figure S4.** Investigation of protein coding genes which are common and unique for CO and COm cells.

## Data Availability

The datasets used and analyzed during the current study are available from the corresponding author on reasonable request.
