# Supplementary material for: MLL-AF4 and a murinized pSer-variant thereof are turning on the nucleolar stress pathway
Source: Cell Biosci. 2022 Apr 25;12:47. doi: 10.1186/s13578-022-00781-y (PMC9036721; doi:10.1186/s13578-022-00781-y)
Supplement: Supplementary file 13 — Additional file 13: Figure S1. Bioinformatic analysis of MACE-Seq data-I Heatmaps analysis. Figure S2. Bioinformatic analysis of MACE-Seq data-II Volcano plot analysis. Figure S3. Investigation of protein coding genes which are common and unique for MA4 and MA4m cells. Figure S4. Investigation of protein coding genes which are common and unique for CO and COm cells. [file 13578_2022_781_MOESM13_ESM.pdf]

Supplemental Data file

Supplemental data files (Excel):

The first set of 5 files (MACE-data) contains the gene entries with gene identifier, gene symbol, gene name, mean exp, mean mock, fold change log2, pvalue, -log10(pvalue), var-log2, chromosome number (with beginning and end), classification (into pseudogene (PG), non-annotated gene (NA), LINC RNA gene (LINC), MIR gene (MIR), SNO gene (SNO), MT gene (MT) and protein coding gene (PCG)).

Data sets 6 and 7 contain the data used for heatmap analysis.

Data sets 8-12 contain the data used for volcano plot analysis

1. MA4 gene signature.xlsx
2. MA4m gene signature.xlsx
3. A4M gene signature.xlsx
4. MA4_A4M gene signature.xlsx
5. MA4m_A4M gene signature.xlsx
6. Heatmap dataset 1 xlsx
7. Heatmap dataset 2 xlsx
8. MA4 volcano.xlsx
9. MA4m volcano.xlsx
10. A4M volcano.xlsx
11. MA4_A4M volcano.xlsx
12. MA4m_A4M volcano.xlsx

Supplemental Figure legends:

Figure S1: Bioinformatic analysis of MACE-Seq data-I

Heatmaps analysis. MACE experiments were performed and resulting data were analyzed by Bioconductor software to create output Excel files. Various bioinformatic tools were used to analyze these data: heatmaps with http://www.heatmapper.ca/expression/. Only protein coding genes have been analyzed.

Figure S2: Bioinformatic analysis of MACE-Seq data-II

Volcano plot analysis. MACE experiments were performed and resulting data were analyzed by Bioconductor software to create output Excel files. Various bioinformatic tools were used to analyze these data: volcano plots with huygens.science.uva.nl. Only protein coding genes have been analyzed.

Figure S3: Investigation of protein coding genes which are common and unique for MA4 and MA4m cells

From the MACE-Seq data, all protein coding genes were extracted that are expressed only in MA4 or MA4m cells. These gene sets (log2 ± 1) were separated for up- and down-regulated genes and then analyzed for overlapping and idiosyncratic genes. As displayed, MA4 and MA4m share 7 commonly up-regulated genes, while 44 and 61 uniquely up-regulated genes, respectively. Similarly, MA4 and MA4m share 26 commonly down-regulated genes, while 79 and 76 uniquely down-regulated genes, respectively.

Figure S4: Investigation of protein coding genes which are common and unique for CO and COm cells

From the MACE-Seq data, all protein coding genes were extracted that are expressed only in CO or COm cells. These gene sets (log2 ± 2) were separated for up- and down-regulated genes and then analyzed for overlapping and idiosyncratic genes. As displayed, CO and COm share 18 commonly up-regulated genes, while 84 and 18 uniquely up-regulated genes, respectively. Similarly, CO and COm share 7 commonly down-regulated genes, while 112 and 14 uniquely down-regulated genes, respectively.

Figure S1

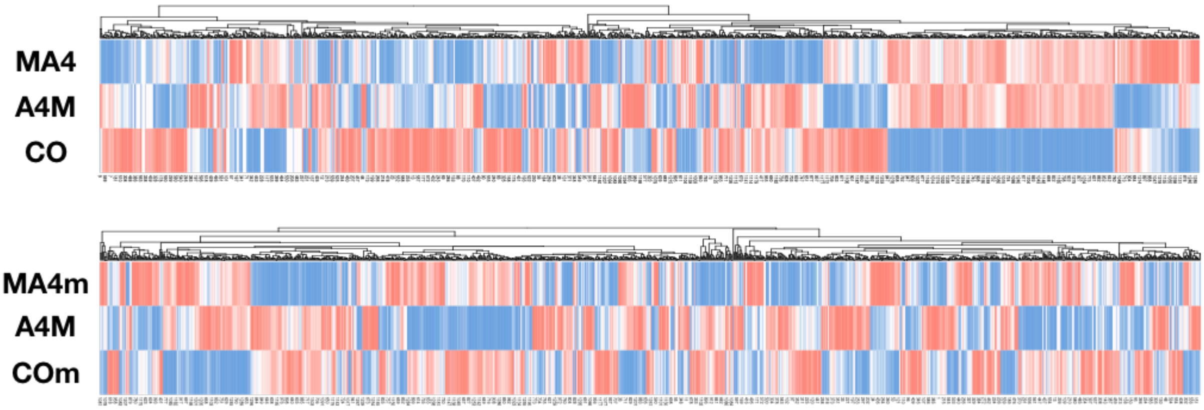

Figure S2

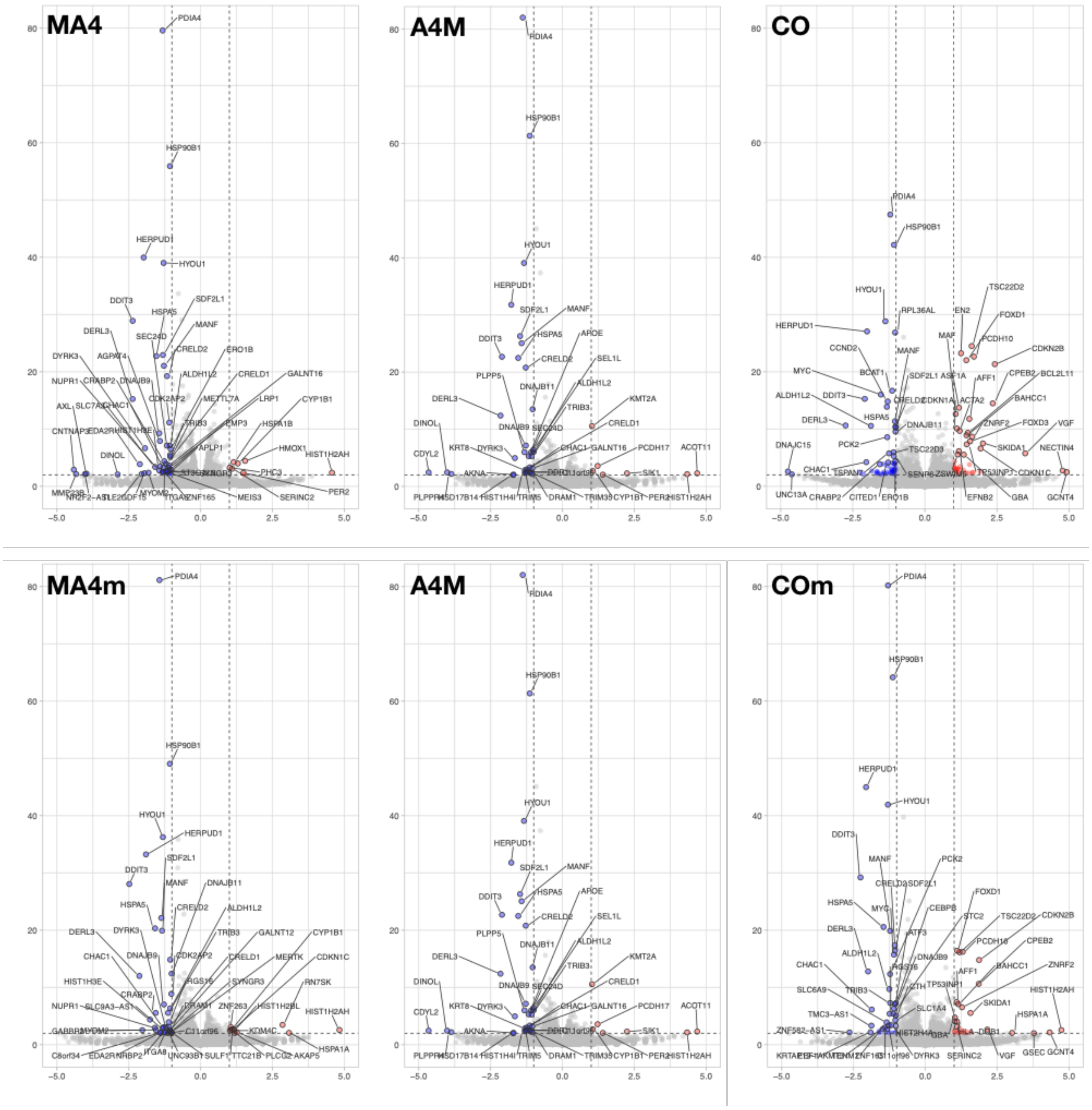

Figure S3

common and unique PCG genes in MLL-AF4 and MLL-AF4m (>2 -fold) completely absent in A4M/Co/Com

>2 fold

IGSF9	ZP3
HIST1H2BG	TAF7L
ZNF852	NPIPA5
CPZ	PTAFR
KCNB1	VAC14-AS1
RNA5S1	RHF12
KCNQ5-IT1	SGK1
BORCS8-MEF2B	HEXA-AS1
FRMD6-AS1	HSPA1B
C4A	C18otf65
CLIC1	ACTRT3
ZNF670-ZNF695	JSRP1
HMOX1	PEX6
SLC26A4-AS1	PDE1A
TEX14	CCDC88B
**FOXN4**	KCNB2
ZNF331	PHDA1
SGK494	ISG20
ADAMTS17	ZSCAN20
RNVU1-7	MAP1LC3B2
RNU4-1	SOX2-OT
ST6GALNAC	HIST2H3D

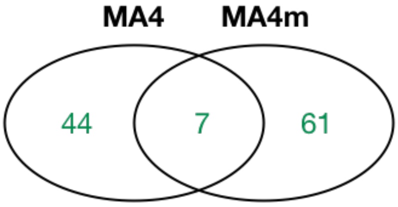

FAM223B
OR2AG2
IGSF10
CLPSL2
EVA1C
GLI1
HIST1H1E

>2 fold

AKR7A3	SLC5A5
ADAM12	SND1-IT1
SOWAHD	UBE2L5
RN7SK	HIST1H2BL
TEMEM35A	PF4
TKTL1	DCDC1
HSF4	CASC1
DPPA4	RASAL2-AS1
C9orf43	AVIL
TNNC1	COL19A1
MIAT	SPDYA
LRRC9	RUBCNL
SCN4A	SCARF2
NPFFR2	ABCA8
GLDN	ZNF597
B3GNT8	GBX2
KCP	FALEC
ABI3BP	MAP2K14
**CD72**	IQCH
GPR61	NKX1-2
ZNF429	COL26A1
GRM3	**PBX2**
ZNF724	LRRC27
RNU1-4	ZNF263
**HOXA6**	ARF5
PDE3A	TT21B
CACNA1C	RTN41P1
C12orf60	**VAV3**
RNU1-2	RHDF2
PLCG2	HLA-DOB
**KDM4C**	

common and unique PCG genes in MLL-AF4 and MLL-AF4m (<2 -fold) completely absent in A4M/Co/Com

>2 fold

PRP15	FAM27B	SLC2A3
KLHL41	ZDHHC11B	SEC14L2
IL17RC	TEF	DACT3
DZANK1	FAM183A	CHODL
ADAMTS7	RHCE	CNIH3
ERICD	ST3GAL3	C1orf54
CCR10	**MEIS3**	SLC9A9
MTRNR2L3	**SOX3**	APLP1
KCNN1	MAP3K12	HIST2H3C
PRPH	**RUNX1**	
CCDC148	CCDC163	
RYR2	SAMD14	
SLC7A3	C8orf37	
KREMEN2	LRRC24	
PDX1	OBSCN	
RAB6C-AS1	SCN9A	
SCNN1D	KCNK15-AS1	
PRKAR2A-AS1	ZSWIM9	
LSAMP	TFEB	
NUAK2	LRP1	
CSDC2	MYT1	
PI15	GUCY1A2	
WIPI1	NLGN1	
**HES2**	PVT1	
GSDMB	PROX1	
DLX6-AS1	LRRC29	
ACAP1	HASPIN	
IRS4	METTL7A	
OPHN1	MMP2	
KCNAB1	ABTB1	
**RAD51**	RAD21-AS1	
CASKIN1	NHLH2	
ZNF883	HIST1H2BC	
RNU4-2	SRRM3	
GEM	PHOSPHO1	

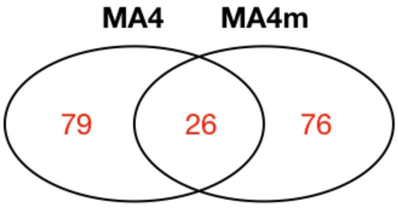

PTPRNA
FBOX2
EDA2R
CPB2-AS1
C5orf49
DENND6B
TNFSF13B
EPHA6
DYNC1I1
HIST1H3E
SECTM1
ITGA8
FBXL22
STKLD1
HNF4G
WDR78
NRBP2
MELTF
RASA4
SLC12A5-AS1
TENM4
CDK2AP2
CRLF1
PLD5
UNC63B1
PANO1

>2 fold

SULF1	RNU6-2	CPLANE2
PPP1R16B	HIST1H4H	FAM201A
TEX22	TMEM31	BDNF-AS
SLC9A2	**PAX1**	PTGER2
ERVK13-1	SORBS1	ARHGEF19
MERTK	CMTM1	SLC6A16
ADPRM	TNFAIP2	OXTR
APBB3	ZNF559-ZNF177	
INAVA	AARD	
ZNF619	NAIP	
KCNH2	MROH6	
CPNE7	SLC9A3-AS1	
ZNF91	SERPINB9	
GALNT12	NHSL2	
C16orf71	STX18-AS1	
COL4A4	GPR135	
AGAP9	AFDN-DT	
SPHK1	C8orf34	
SYT16	TENT5B	
ARHGAP24	**DLL4**	
SLC44A3	CEACAM19	
**DLK2**	PRDM8	
ZNF365	FOXD4L1	
**FOSL2**	CRIP3	
C10orf25	GABBR2	
OSBPL10	MST1	
CEBPD	IL20RB	
PIGZ	FAM189A2	
RTCA-AS1	GALNT3	
TMEM74B	SPNS2	
DAB1	CA13	
**FOSB**	KDM4A-AS1	
SPON2	IGSF21	
CACNA1B	SLC26A10	
C1QL4	ST6GALNAC5	

Figure S4

common and unique PCG genes in CO and COm (>4 -fold) absent in MA4, MA4m, A4M

>4 fold

TCHH
FAM238C
ETV7
MYO15A
CXorf58
HHIPL2
C20orf204
RNA5S17
B4GALT1-AS1
NUTM2D
IFIT2
C5orf66
CCDC116
KCNK9
RS1
CYBB
CSRP1
MSR1
ADNP-AS1
FOXL1
SPOCK1
ASGR1
DDC
SULT2B1
LYPD3
STAT4
C1RL
SULT1C4
RORA-AS1
FILIP1
HYMAI
NEUROD2
TM6SF1
ADAMTS10
CDKN2B

CBLN1
NRROS
TLL2
CPEB2
AFAP1L2
VSIG2
ZNF460
FOXQ1
ZNF154
ASIC3
PCDHA10
PLXDC1
NRK
C2CD4A
FMNL1
FGF21
ADAMTSL3
CLDN23
SLC8A2
SDR42E2
PARD3-AS1
LY6K
GPR1
SOCS3
FRRS1
ST8SIA5
IL1R1
DUSP5
DENND2A
P2RX2
DDX39B-AS1
ANXA9
IL32
DHX58
MAGEA6

DUSP15
TPH1
MYO18A
ADAMTS2
TMED6
ZNF816
PIFO
ESAM
ATP8A2
AMER2
SLC24A2
SKIDA1
DENND1C
DND1

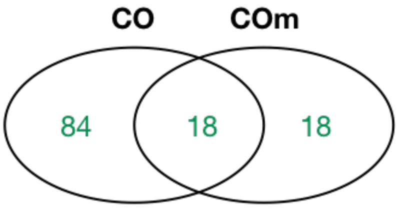

ZC2HC1C
CALHM5
CFAP221
STYK1
VGF
AQP4
UNC5B-AS1
SFN
CCDC39
PVALB
TMEM86B
PDGFRA
GRIN2C
DQX1
FAM66C
ETV5
CHRNB4
BTBD19

>4 fold

WBP2NL
PDE1B
TSPOAP1-AS1
EDRF1-AS1
TEKT3
FAM181B
TMEM191B
KLHDC7A
TNFRSF25
TSPAN19
HLA-DMA
KBTBD11-OT1
BRICD5
KYNU
DUSP6
BDKRB2
TFCP2L1
ARHGEF37

common and unique PCG genes in CO and COm (>4 -fold) absent in MA4, MA4m, A4M

>4 fold

DNAJC15
UNC13A
PLIN5
TMEM132E
N4BP2L1
C9orf139
KLHL32
TGFB2
COL9A2
KIAA0825
AMIGO2
GDF6
IRX6
CLUL1
MTMR7
IKZF3
IGSF11
SLC26A1
CD68
TMEM217
PPP1R27
TMEM202-AS1
PRRT1
LRRK2
TMEM225B
SOCS2-AS1
VSTM2L
BMP2
PAUPAR
SKOR1
ART4
MATN4
SNED1
CD44
EPHA5
ATG16L2
MCF2L

ABCB4
PCDHB13
FUT1
NOTCH4
DLX3
SLC11A1
THSD7A
SEMA3B
FHAD1
PTGFR
CHST1
CDKN2A-DT
TLE6
TBC1D3H
PSD
URAD
C6orf141
DDX58
BTG3-AS1
S100A4
ANKDD1A
TMEM221
SULT1A3
RAB39A
BCDIN3D-AS1
TBX19
CCBE1
TBC1D8
PCDH19
ITGA4
MYOD1
FGF12
VIPR1
ADAMTS6
SLC10A5
ENO1-AS1
CLMP

HSFX3
ENO3
POC1B-AS1
ARAP3
CALHM2
SH3D21
CPT1B
CXXC4
CCDC110
PTHLH
PDCD4-AS1
C10orf82
HIST3H2BB
HMGN4
KCNS3
EPHA5-AS1
TEKT2
TERT
JAM2
FAM169B
FAM167A
TOB1-AS1
KCNQ1
EDNRB
MAPK13
PILRB
C11orf65
H2BFS
ARHGAP8
RAB41
CYP1A1
C15orf48
CHAC1
OSBPL7
INHBE
SP2-AS1
LRMDA
TMEM63C

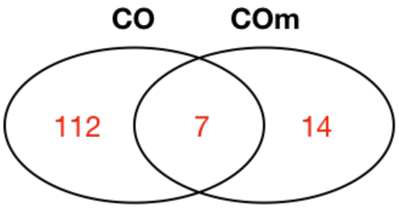

KCNIP4
FGF7
TGFB2-AS1
TRHDE
DIRAS3
HCN4
HERPUD1

>4 fold

OSTN
PTF1A
ZNF582-AS1
UBAP1L
PNMT
GPSM3
ZNF596
ST7-AS1
ODF3L2
CEBPB-AS1
NDUFA4L2
ERBB4
IL18BP
NPPA-AS1
